# Empirical formula for the prediction of off axis ratios and isodose curves for a treatment planning system

**DOI:** 10.4103/0971-6203.29196

**Published:** 2006

**Authors:** Surajit Pal, R. Ravishankar, R. P. Sharma, G. Muthukrishnan, Dilip Kr Ray, S. N. Roy, D. K. Srivastava

**Affiliations:** Variable Energy Cyclotron Centre, 1/AF Bidhan Nagar, Kolkata, India; 1 Health Physics Unit, VIP Road, Kolkata, India; 2 L - 404 VIP Enclave, VIP Road, Kolkata, India; 3 B-12, Adhunika Co-operative, 164/3A Lake Gardens, Kolkata, India; 4 Chittaranjan National Cancer Institute, Kolkata, India; 5 Department of Physics, Visva-Bharati, Santiniketan, India

**Keywords:** Isodose curves, off-axis ratio, percentage depth dose, treatment planning

## Abstract

A mathematical model has been developed for prediction of off axis ratio (OAR), using Wood - Saxon term used to represent nuclear potential. This method has been satisfactorily applied for predicting OAR in case of ^60^Co γ-rays and high energy X-rays. Investigations are considered upto a depth of 25 cm in the case of 4MV LINAC for which measurements were carried out in our laboratory using indigenously developed Radiation Field Analyzer. For ^60^Co γ-rays as well as 6 and 18MV LINAC beams we could get off-axis profiles only upto 20 cm. The shift δ between measured and predicted OAR is within ±2 mm except for 20 cm depth near the falling edge of the penumbra, where it is 2.80 mm. Software has been developed in Visual Basic 6 on Windows platform to plot Isodose curves, which is based on the mathematical modeling of OAR and central axis percentage depth dose.

The use of empirical formula for computing doses along the central axis and off axis ratio (OAR) along the transverse axis is advantageous in many ways. It reduces the databank requirements, which leads to a decrease in number of measurements to be made. Since one can predict the dose distributions for any field size, rectangular field sizes present no problem. In the case of high-energy x-ray machines, the shape of OAR curves and hence Isodose curves will depend, to a large extent, on the design of the beam flatteners.[1] It is well recognized among the clinical medical physicists that published Isodose curves can not be used and that the dose calculation performed must be specific to the teletherapy machine used, therefore, empirical formulations become quite necessary. The empirical formulations, so developed, should be able to reproduce the physical situation with a reasonable accuracy. It should also be possible to adopt the formula for any treatment machine. By reasonable accuracy we mean the distance in millimeters measured along the transverse axis between the points where the measured and calculated doses are same. According to the work of Thomas[2] and Khan,[3] a maximum shift of 2 mm is taken as sufficiently accurate, which we have adopted.

Various methods have been developed for the prediction of off axis ratios.[2,4,5] Usually in all these formulations, two or three sets of formulae are used to cover the penumbra and umbra regions etc. Kornelson[4] suggested the use of Fermi-Dirac distribution function[6] to represent the OAR in the case of moving field treatment (SAD technique). But when we applied the same for fixed field cases (SSD technique) it could not be fitted to the Fermi-Dirac Distribution. We have, therefore, developed a method in our laboratory based on Wood-Saxon term,[7] which is generally used to represent nuclear potential. Here, a single formula can predict the off axis distributions covering the whole region. The method is based on the formula, developed for ^60^Co therapy beam by Ravishankar[8] and we extended the same for the case of 4MV LINAC and other high-energy machines, thus demonstrating the versatility of the method.

In order to arrive at a suitable treatment plan, we need both central axis percentage depth dose and off axis ratio. In the earlier publication,[9] the method to calculate central axis percentage depth dose (CAPDD) using buildup concept has been explained. We present here the method to calculate OAR and also the development of treatment plan.

## Materials and Methods

The OAR is the ratio of off-axis dose to the central axis dose at the same depth. The plot of OAR *vs.* transverse distance resembles the formulation of Wood - Saxon term, extensively used in nuclear physics.[7] The off axis ratio *R* can be expressed as

(1) $$R=\frac{1}{\left[1+\text{exp }\left\{wx\left(\times \text{-}x_{0}\right)\right\}\right]}$$

where *x*_0_ is the half width of the square field at depth *d* below the surface of the water medium and is given by

(2) $$x_{0}=\left(\frac{n}{2}\right)\times \left(\frac{f+d}{f+d_{m}}\right)$$

and *x* is the off axis distance, *f* is the SSD, *n* is the field width at the depth of maximum dose (*d_m_*). We have tested Eqn. (1) only for square fields. One has to study the validity for rectangular fields. The factor *w* is expressed as follows:

(3) $$w=\frac{1}{\text{S}}{\left(\frac{f\text{-}f_{c}+d}{f_{c}}\right)}^{k}$$

where *S* is the source diameter and *f_c_* is the source to collimator distance. For a given depth and field size, *w* should be strictly constant. But due to experimental errors or constructional details in the beam flattener, *w* varies with *x*. As in the studies with central axis depth dose distributions,[9] it is always possible to find a value of *w*, which will fit the OAR to the accuracy mentioned earlier. This is the basis of the formalism.

**Figure 1:**
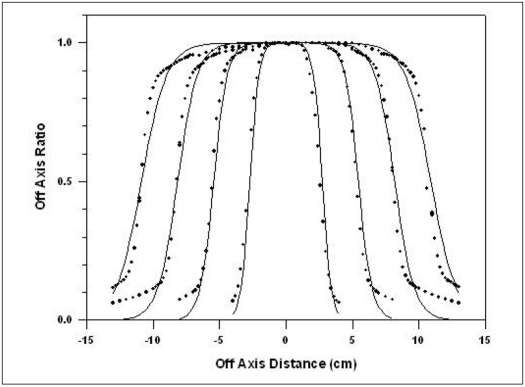
Comparison of measured and calculated OAR values for the field sizes of 5×5, 10×10, 15×15 and 20×20 cm^2^ at 10 cm depth for 4 MV Medical LINAC Jeevan Jyoti 2. The line represents the calculated values whereas dots are measured values

From Eqn. (1) we find the values of *w* for different OAR's. From these values of *w*, a suitable weighted average value of *w* is chosen so that it fulfills our requirement for the reproduction of OAR's. The *w* value was weighted over the OAR values, *w*(weighted) =ɛ*w* x *R*/ɛ *R*. The *w* values at *x* = x_0_ or *x* close to *x*_0_ are omitted as *w* →∞. Using this value of *w*, *R* was calculated for field sizes 5×5, 10×10, 15×15 and 20×20 cm^2^ and for depths 5,10, 15 and 20 cm. The required data for all the field sizes and depths were taken from the measurements made at 4MV Medical LINAC Jeevan Jyoti-2.[10] Figure 1 shows the comparison between measured and calculated values for the various field sizes at a depth of 10 cm. Table 1 gives the shift (δ mm) along the off axis between the points and position where the measured dose and the calculated dose are the same. Measurements are carried out in a water phantom (Radiation Field Analyzer) using 0.125cc, PTW make, semiflex ionization chamber. Depth of maximum dose is 1 cm for 4 MV x-ray beam. It is clear from Table 1 that δ lies within 2 mm.

**Table 1:** Shift (δ) along the off axis between calculated and measured OAR for 4MV LINAC in the water phantom

*Depth (cm)*	*Measured OAR*	*5 × 5 cm^2^ field*			*10 × 10 cm^2^ field*		

		*Calc. OAR*	*δ (mm)*	*Off axis distance (cm)*	*Calc. OAR*	*δ (mm)*	*Off axis distance (cm)*
5	1.0000	1.0000	-	0	1.000	-	0
	0.971	0.97	0.10	−1.45	0.975	0.7	3.40
	0.968	0.907	0.10	−1.48	0.963	0.83	−3.60
	0.883	0.882	0.02	−1.93	0.837	1.90	4.40
	0.715	0.693	0.36	−2.33	0.693	0.55	4.80
	0.586	0.555	0.43	−2.53	0.600	0.28	5.00
	0.512	0.509	1.80	−2.59	0.499	0.85	−5.20
	0.444	0.413	0.42	−2.72	0.499	1.10	5.20
	0.385	0.356	0.39	−2.79	0.398	0.28	5.40
	0.269	0.249	0.25	2.97	0.305	1.04	5.60
	0.1	0.083	0.59	−3.39	0.114	0.80	6.20
	0.087	0.070	0.80	−3.46	0.079	0.46	6.40
20	1.000	1.000	-	0	1.000	-	0
	0.974	0.972	0.26	1.69	0.978	1.00	3.60
	0.931	0.944	0.76	1.95	0.924	0.70	4.40
	0.825	0.843	0.57	2.36	0.821	0.02	5.00
	0.759	0.767	0.18	2.54	0.769	0.32	5.20
	0.68	0.671	0.15	2.71	0.706	0.73	5.40
	0.588	0.550	0.55	2.90	0.635	1.21	5.60
	0.478	0.419	0.88	3.09	0.557	1.95	5.80
	0.294	0.212	1.50	3.45	0.396	2.80	6.20

OAR - Off axis ratio

For 4MV Medical LINAC of RRMC, *f* = 100 cm, *f_c_* = 38 cm and *S* = 0.2 cm. Table 2 gives the *w* values (calculated and fitted) as a function of depth for a field size 10×10 cm^2^.

**Table 2:** Calculated and fitted values of *w* for 10 × 10 cm^2^ field size for various depths. (4MV from Jeevan Jyoti-2 and 6 and 18MV from Siemens LINAC)

*Depth (cm)*	*4 w (cal)*	*MV w (fitted)*	*6 w (cal)*	*MV w (fitted)*	*18 w (cal)*	*MV w (fitted)*
5	2.12	1.97	3.44	2.61	2.39	2.29
10	1.98	1.88	2.32	2.45	2.19	2.16
15	1.72	1.8	-	-	-	-
20	1.62	1.73	2.28	2.18	1.86	1.96
25	1.5	1.66	-	-	-	-

The *k* values are again fitted against field sizes by the following equation

(4) k=axln(n)+b

where *n* is the field size and *a* and *b* are constants. For our case *a* = -1.127 and *b* = 1.0544.

Hence using Eqs. (3) and (4) we can estimate *w* value for any field size and depth to predict OAR. This enables us to generate isodose curves as explained in the sub-section Isodose Curves.

### 6 and 18 MV x-rays

**Figure 2:**
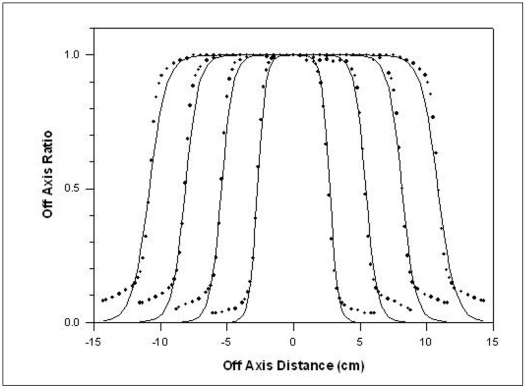
Comparison of measured and calculated OAR values for the field sizes of 5×5, 10×10, 15×15 and 20×20 cm^2^ at 10 cm depth for 6 MV x-rays. The line represents the calculated values whereas dots are measured values

**Figure 3:**
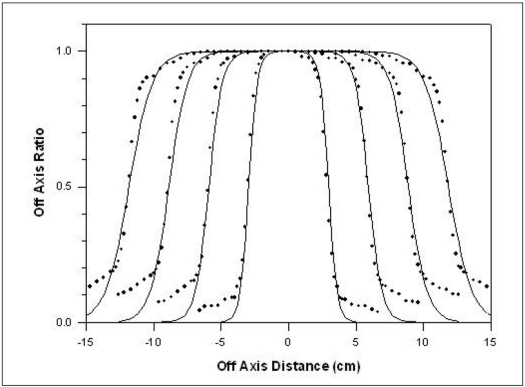
Comparison of measured and calculated OAR values for the field sizes of 5×5, 10×10, 15×15 and 20×20 cm^2^ at 20 cm depth for 6 MV x-rays. The line represents the calculated values whereas dots are measured values

**Figure 4:**
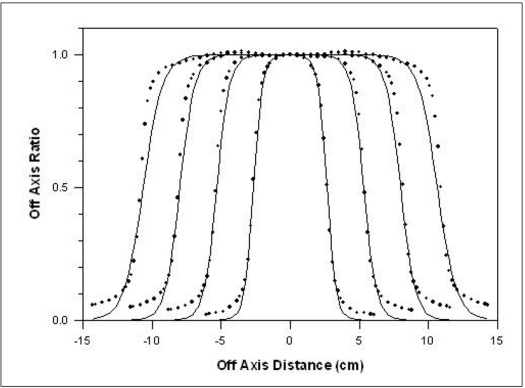
Comparison of measured and calculated OAR values for the field sizes of 5×5, 10×10, 15×15 and 20×20 cm^2^ at 10 cm depth for 18 MV x-rays. The line represents the calculated values whereas dots are measured values

The method was applied in the OAR of 6 and 18MV X-rays produced from Siemens LINAC (Mevatron, KD-2), installed at the Chittaranjan National Cancer Institute, Kolkata for various field sizes. Depths of maximum dose is 1.4 and 3.4 cm for 6 and 18 MV x-ray beam, respectively. For Siemens LINAC (Mevatron, KD-2), *f* = 100 cm, *f_c_* = 39.2 cm and *S* = 0.2 cm. Table 3 gives the value of parameters *a* and *b* for 4, 6 and 18 MV x-rays as calculated by using Eqn. (4). Figures 2–4 show the comparison between measured and calculated values of OAR for various field sizes for 6 and 18 MV x-rays. Table 2 gives the *w* values (calculated and fitted) as a function of depth for a field size 10×10 cm^2^ for these energies.

**Table 3:** Values of constants *a* and *b* of Eqn. (4) for different beam energies

*X-ray energy (MV)*	a	b
4	−1.127	1.0544
6	−0.9107	0.8522
18	−0.8967	0.6832

### ^60^Co source

We have also applied the method for ^60^Co Teletherapy machine Picker C-2000 unit. The necessary input is taken from Van de Geijn[11] for a field size of 10×10 cm^2^ for various depths. Fgirues 5 compares the typical measured and the calculated OAR values for a depth of 5 cm and the agreement between the two is quite reasonable. In this case the values are fitted by the following relationship.

(5) $$w=\text{A}\times {\left(\frac{f\text{-}f_{c}+d}{f_{c}}\right)}^{k}$$

Where *A* is 1.4736 and *k* is -1.0866. The values of *f* and *f_c_* considered for the calculation are 50 and 27 cm, respectively.

Table 4 gives the calculated and fitted values of *w* for various depths.

**Table 4:** Calculated and fitted values of *w* for ^60^Co beam, field size 10 × 10 cm^2^(*k* = -1.0866, *A* = 1.4736)

*Depth (cm)*	w *calculated*	w *fitted*
2	1.62	1.60
5	1.42	1.42
10	1.12	1.18
20	0.93	0.89

### Isodose curves in water medium

From the literature it is observed that the Decrement Line Method[12] and Fan-Line Method,[13] are used to generate isodose curves. However, we have used another approach where a semi-empirical analytical method is applied to generate isodose curves. The basic equations used are given below.

In general, total dose *D* is represented by[14]

(6) $$D=B\times D_{0}$$

where *B* is the dose buildup factor and *D*_0_ is the contributions from the primary radiation. *B* is given by:

(7) B=1+s

*s* represents the ratio of scattered to incident primary radiation,

We have used the same approach to represent the Central Axis Percentage Depth Dose (CAPDD)[9] in the same form as follows:

Total dose *D*_1_ at a depth of *d*_1_ cm beyond depth of maximum dose is given by:

(8) $$D_{1}=D{\text{0}}_{1}\left(1+s_{1}\right)$$

Total dose *D*_2_ at depth *d*_2_ cm (depth of maximum dose) is given by:

(9) $$D_{2}=D{\text{0}}_{2}\left(1+s_{2}\right)$$

where *D*0_1_ and *D*0_2_ are primary dose contributions and *s*_1_ and *s*_2_ are scattered components.

∴ Relative dose

(10) $$D=\frac{D_{1}}{D_{2}}=\frac{D{\text{0}}_{1}\left(1+s_{1}\right)}{D{\text{0}}_{2}\left(1+s_{2}\right)}=D_{0}\frac{\left(1+s_{1}\right)}{\left(1+s_{2}\right)}$$

where

$D_{0}=\frac{D0_{1}}{D0_{2}}$

This can be approximated to

(11) $$D=D_{0}\left(1+s\right)$$

where

$S=S_{1}\text{-}S_{2}{\text{-S}}_{1}S_{2}$

since *s*_1_ and *s*_2_ are relatively small.

*D*_0_ corresponds to the dose value of 0×0 cm^2^ field and is obtained by extrapolation of measured/published values of percentage depth doses for different depths. D_0_ values at different depths are found to decrease exponentially.

Now

(12) $$s=a_{1}\times r^{kl}$$

*s* represents the ratio of scattered to incident primary radiation, *r* is the depth inside the water phantom expressed in terms of mean free paths; *a*_1_ and *k*_1_ are constants. Equations (11) and (12) are valid for all depths greater than the depth of maximum dose. Also in the energy range considered there is no appreciable difference between the kerma and the absorbed dose.

The off axis distance, corresponding to the required OAR from Eqn. (1) is given by:

(13) $$x=x_{0}+\left[\frac{1}{w}\right]\text{xln}\left(R^{'}\text{-1}\right)$$

**Figure 6:**
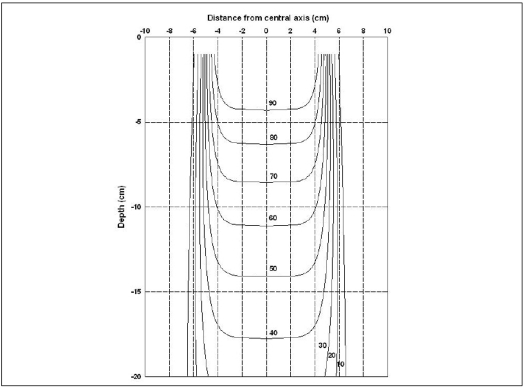
Isodose curve in water medium of 4MV x-rays from Jeevan Jyoti 2 for 10×10 cm^2^ field

**Figure 7:**
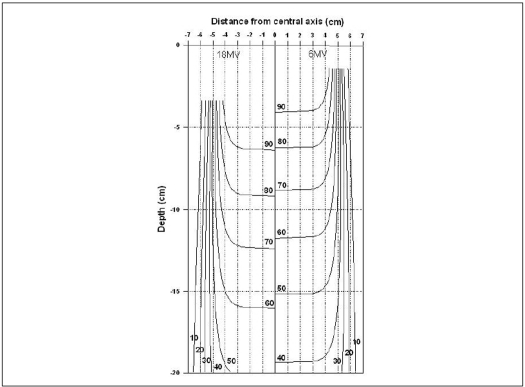
Isodose curves in water medium of 6 and 18MV X-rays from Siemens LINAC for 10×10 cm^2^ field

Here *R'* is the inverse of OAR, which is normalised w.r.t. the percentage depth dose ratio along the central axis at the required depth using Eqn. (11). Eqn. (13) is valid for single fixed field only. We generated (x,y) coordinates for 10×10 cm^2^ field size and various percentages (5–90%). Figure 6 shows the Isodose curves for the 4MV x-rays and [Figure 7] gives the same for 6 and 18 MV x-rays.

**Figure 8:**
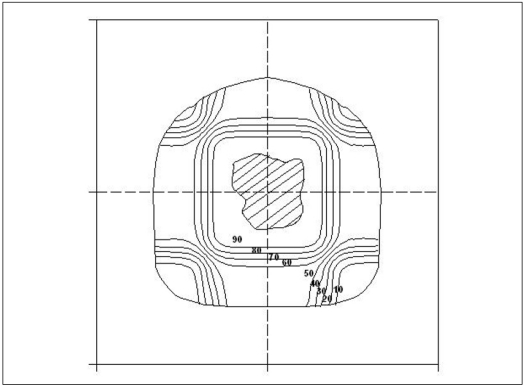
Multiple fields (four orthogonal, 10×10 cm^2^ field) Isodose distributions of 4MV x-rays from Jeevan Jyoti 2

The total dose distributions for multiple fields treatment plan is obtained by calculating doses at each grid point of a matrix. The total area considered has been divided into grids with Cartesian coordinate system. The dose at the grid point, with a grid spacing of 0.2 cm, was then calculated from Eqs. (1) and (11), which give OAR and CAPDD, respectively. This procedure was repeated for all the fields for a given treatment plan and the total dose at each grid point was obtained by summing up the doses and then it was normalized against the maximum dose in percentage. Figure 8 represents a typical treatment plan with an arbitrary patient contour and target for four orthogonal fields of 10×10 cm^2^, where shaded portion represents the target volume.

Contour corrections are also included in this planning system. This is obtained by finding out air gap/extra tissue thickness due to shape of the contour and then multiplying by a suitable correction factor to each grid point.[15,16]

### Computer program

A computer program is developed in Visual Basic 6 on Windows platform to process the data faster and plotting of OAR and isodose curves. The algorithm is as follows:

- Reading of measured OAR, off axis distance, field size, depth of maximum dose, source to surface distance, depth of measurement, source to collimator distance and source diameter, if applicable.
- Calculation of *w* for each depth and field size.
- Least square flitting of *w* with depth to calculate the constant *k* for each field size.
- Least square fitting of *k* with field size in the Eqn. (4) to find the constants *a* and *b*.
- Finally, the values of *a* and *b* are used to calculate OAR for any field size and depth.
- For ^60^Co beam least square fitting of *w* with depth is used to calculate not only *k* but also *A*, another constant from Eqn. (5).
- Knowing the above constants OAR's are calculated. Similarly, Central Axis Depth Doses are calculated through the Eqn. (11). The product of these two quantities gives the percentage depth dose for each grid point.
- Dose for each grid point is plotted in the ‘Picture Box’ to generate and display isodose curves.
- Treatment planning for multiple fields is executed by summing up dose values at each grid point from each field and the same is normalized against the maximum dose.

## Results and discussion

The factor *w* should be constant for a particular depth and field size for all off axis distances. But in actual practice it has been observed that *w* changes with off axis distance. To avoid any complexity, weighted average of *w* is taken and this takes care of variations of both *w* and OAR. In our experiments *w* is calculated for three depths for 6 and 18 MV x-rays and five depths for 4 MV x-rays. In both the measurements good agreement between calculated and measured OAR is obtained.

**Figure 5:**
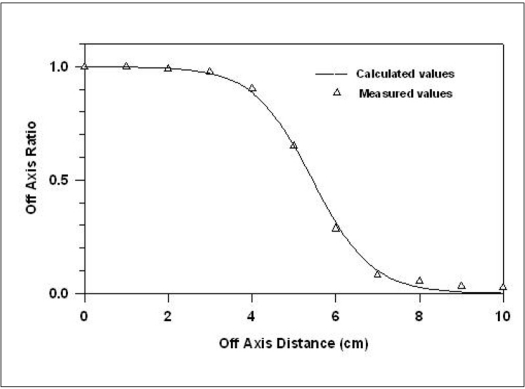
Comparison of measured and calculated OAR values for ^60^Co Teletherapy Unit (Picker C-2000) for a field size of 10×10 cm^2^ at 5 cm depth. (Measured data taken from Van de Geijn)[11]

Referring to Figures 1–5, the agreement between the measured and calculated OAR in the central region is within ±2%. In the penumbra region, the shift in the particular OAR is within 2 mm. We used this criterion of Khan[3] as this satisfies all field sizes and depths.

**Figure 9:**
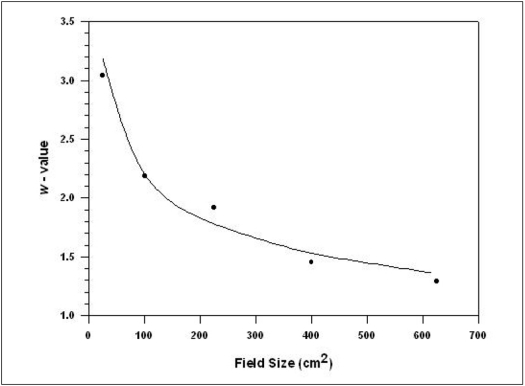
Comparison between calculated and fitted values of *w* with field size for 18 MV x-ray beam at 10 cm depth. Field size is represented in terms of equivalent square. The line represents fitted values whereas dots are calculated ones

The fitted and calculated values of *w* are shown in the Table 2, it is clear that the variation between fitted and calculated value is quite significant. However, this is not affecting the final result of off axis ratios, adversely. Figure 9 is the graphical representation of actual and fitted values of *w* with respect to field size for 18 MV x-ray beam at 10 cm depth. Table 5 gives the percentage deviation in dose along central axis between the isodose calculated by our algorithm and the same from a commercial planning system. Comparison of 4 MV x-rays could not be considered since commercial values for 4 MV are not available locally.

**Table 5:** Deviation in percentage dose along central axis, calculated with our algorithm and with a commercial TPS

*Depth (cm)*		*6MV X-rays*			*18MV X-rays*	
	Our algorithm	Commercial TPS	Percentage variation	Our algorithm	Commercial TPS	Percentage variation
5	85.42	86.35	1.08	95.89	95.81	−0.08
10	66.2	66.09	−0.17	77.98	77.91	−0.09
15	50.72	50.24	−0.96	63.30	63.14	−0.25
20	38.15	38.04	−0.29	50.99	51.07	0.16

TPS - Treatment planning system

## Conclusion

This is a simple technique for the prediction of off axis ratios, which is finally used for the development of treatment planning system along with mathematical modeling of central axis percentage depth dose technique developed earlier.[9] The system can be used by the cancer centers of our country that do not have access to sophisticated treatment planning systems due to high cost and maintenance. The system was evaluated at the Chittaranjan National Cancer Institute, Kolkata and the same is found to be accurate and user friendly. The software in the present form does not include corrections for heterogeneity of cancer patients as well as for irregular fields.
